# The lipidome in nonalcoholic fatty liver disease: actionable targets

**DOI:** 10.1016/j.jlr.2021.100073

**Published:** 2021-05-05

**Authors:** Carlos J. Pirola, Silvia Sookoian

**Affiliations:** 1 Instituto de Investigaciones Médicas A Lanari, Facultad de Medicina, Universidad de Buenos Aires, Ciudad Autónoma de Buenos Aires, Argentina; 2 Departamento de Genética y Biología Molecular de Enfermedades Complejas, Instituto of Investigaciones Médicas (IDIM), Consejo Nacional de Investigaciones Científicas y Técnicas (CONICET)−Universidad de Buenos Aires, Ciudad Autónoma de Buenos Aires, Argentina; 3 Departamento de Hepatología Clínica y Molecular, Instituto of Investigaciones Médicas (IDIM), Consejo Nacional de Investigaciones Científicas y Técnicas (CONICET)−Universidad de Buenos Aires, Ciudad Autónoma de Buenos Aires, Argentina

**Keywords:** lipidomics, NAFLD, NASH, butanoate, microbiome, systems biology, OMICS, PUFA, GPCRs, G protein-coupled receptors, HETE, hydroxyeicosatetraenoic acid, NAFL, simple steatosis, NAFLD, nonalcoholic fatty liver disease, NASH, nonalcoholic steatohepatitis, OxoODE, oxo-octadecadienoic acids

## Abstract

Nonalcoholic fatty liver disease (NAFLD) has become the most prevalent chronic liver disease. Recent technological advances, combined with OMICs experiments and explorations involving different biological samples, have uncovered vital aspects of NAFLD biology. In this review, we summarize recent work by our group and others that expands what is known about the role of lipidome in NAFLD pathogenesis. We discuss how pathway and enrichment analyses were performed by integrating a list of query metabolites derived from text-mining existing NAFLD-lipidomics studies, resulting in the identification of nine Kyoto Encyclopedia of Genes and Genomes dysregulated pathways, including biosynthesis of unsaturated fatty acids, butanoate metabolism, synthesis and degradation of ketone bodies, sphingolipid, arachidonic acid and pyruvate metabolism, and numerous nonsteroidal antiinflammatory drug pathways predicted from The Small Molecule Pathway Database. We also summarize an integrated pathway-level analysis of genes and lipid-related metabolites associated with NAFLD, which shows overrepresentation of signal transduction, selenium micronutrient network, Class A/1Rhodopsin-like receptors and G protein-coupled receptor ligand binding, and G protein-coupled receptor downstream signaling. Generated gene-metabolite-disease interaction networks indicate that NAFLD and arterial hypertension are interlinked by molecular signatures. Finally, we discuss how mining pathways and associations among metabolites, lipids, genes, and proteins can be exploited to infer networks and potential pharmacological targets and how lipidomic studies may provide insight into the interrelationships among metabolite clusters that modify NAFLD biology, genetic susceptibility, diet, and the gut microbiome.

Nonalcoholic fatty liver disease (NAFLD) has become the most common chronic liver disease with global prevalence in the 20–50% range (1, 2). This is in part explained by the co-occurrence of NAFLD with other metabolic diseases and the co-existence of shared risk factors and disease mechanisms between NAFLD and phenotypes of the metabolic syndrome, including obesity, type 2 diabetes, dyslipidemias, and cardiovascular disease (3, 4). NAFLD pathogenesis involves multiple factors, including genetic susceptibility, epigenetic modifications, and diverse interactions with the environment, dietary habits, and sedentary lifestyle (5, 6, 7).

Technological advances that have occurred in the last decade, combined with OMICs experiments and high-throughput explorations across different biological samples, have revealed key aspects of NAFLD biology as well as mechanisms that explain the disease severity.

However, while this field is continuously evolving, how this knowledge can be integrated and applied to biomarker and drug discovery to treat nonalcoholic steatohepatitis (NASH)—the severe histological form of the disease—is less clear. In this review, we explored the potential use of biological data mining and system biology approaches to expand the existing knowledge on the lipidome's role in NAFLD and NASH pathogenesis. Ultimately, mining pathways and associations between metabolites, lipids, genes, and proteins can be exploited to infer networks, interactions, and potential pharmacological targets.

## Lipidomics in NAFLD: Current Knowledge and Clinical Implications

Lipidomics approaches have been used in several human studies to explore the mechanisms that may explain the progression of NAFLD into severe histologic disease stages. A summary of the main studies, including their key findings, is shown in Table 1.

**Table 1:** Summary of human studies on lipid profiling of plasma or liver tissue samples of patients with NAFLD across the spectrum of the disease severity

Author and Reference #	Population Features Sample Size; Liver Histology	Key Finding/s	Analytical Approach
Explorations in plasma or serum			
Puri (8)	Case-control study. Plasma samples of adult biopsy-confirmed NAFLD patients. NAFL (n = 25) and NASH (n = 50); lean normal controls (n = 50)	A stepwise ↑in lipoxygenase (LOX) metabolites 5(S)-hydroxyeicosatetraenoic acid (5-HETE), 8-HETE, and 15-HETE characterized the progression from normal to NAFL to NASH.	HPLC QTRAP. GC-FID
Feldstein (9)	Case-control study. Plasma samples of adult biopsy-confirmed NAFLD patients. NAFL (n = 23) and NASH (n = 37); normal controls (n = 13)	↑ 9-HODE, 13-HODE, 9-oxoODE, and 13-oxoODE, products of free radical-mediated oxidation of linoleic acid in patients with NASH compared with patients with hepatic steatosis and normal liver biopsy.	HPLC-QQQ
Oresic (10)	Individuals in whom liver fat content was measured using proton magnetic resonance spectroscopy or liver biopsy. Discovery (n = 287), validation (n = 392). No information on NAFLD histology	LysoPCs and PUFA-containing phospholipids were negatively associated with liver fat content	UHLC-ToFMS
Zhou (11)	Cases only study of adult subjects who underwent a liver biopsy because of suspected NASH. 318 subjects who underwent a liver biopsy because of suspected NASH. Discovery (n = 223): non-NASH (n = 176); NASH (12). Validation (n = 95): non-NASH n = 71, NASH n = 24.	↑ Saturated and monounsaturated TGs: 21 TG (46:0), TG (48:0), TG (50:0), TG (46:1) and TG (51:1) in NASH.	UPLC-QToFMS GC-ToFMS
Caussy (13)	Cases only study of adult biopsy-confirmed NAFLD patients. 427 patients with biopsy-confirmed NAFLD: NASH with F0–F2 fibrosis n = 229; NASH with advanced fibrosis (F3 and F4) n = 197.	Fibrosis was associated with 11,12-DIHETE, tetranor 12-HETE, adrenic acid, and 14, 15-DIHETE	HPLC-QQQ/QTRAP-MS
Draijer (14)	Case-control study. Plasma sample of obese children with and without fatty liver 21 children with obesity in whom steatosis was detected using proton magnetic resonance spectroscopy (H-MRS).	↑ alkyldiacylglycerol and phosphatidylethanolamine species and ↓ alkyl/alkenyl-phosphatidylethanolamine, alkyl/alkenyl-lysophosphatidylethanolamine and alkyl/alkenyl-phosphatidylcholine	H-MRS
Explorations in liver tissue			
Araya (15)	Case-control study; adult patients. Analysis of liver and abdominal adipose tissue fatty acids 19 patients with NAFLD were studied and 11 control samples. No information on NAFLD histology.	NAFLD tissue has a depletion in LCPUFA (long-chain PUFA) of the n -6 and n -3 series in liver triacylglycerols, ↓ 20:4, n -6/18:2, n -6 and (20:5, n -3+22:6, n -3)/18:3, n -3 ratios, whereas liver phospholipids contained higher n -6 and lower n -3 LCPUFA	GC
Puri (16)	Case-control study. Liver tissue samples of adult biopsy-confirmed NAFLD patients. NAFL (n = 9) and NASH (n = 9); controls (n = 9).	A trend for a progressive ↓ from controls to NAFL to NASH for eicosapentanoic acid (20:5n-3) and docosahexanoic acid (22:6n-3).	TLC GC-FID
Allard (17)	Adult patients referred for elevated liver enzymes and suspected NAFLD. Hepatic FA composition was compared between simple steatosis, NASH and minimal findings on liver biopsy NAFL (n = 18) and NASH (n = 38); controls (n = 17).	↓ hepatic n-3 and n-6 PUFA, ↓in the ratio of metabolites to essential FA precursors for both n-6 and n-3 FA (eicosapentaenoic +docosahexaenoic/linolenic and arachidonic/linoleic acid ratios) and ↑liver lipid peroxides	GC-FID
Garcia-Canaveras (18)	Liver tissue samples obtained from the Liver Bank at the Hospital La Fe (UHE-LAFE/CIBERehd, Valencia, Spain) Nonsteatotic liver n = 23, steatotic liver n = 23. No information on NAFLD histology.	↑phospholipid degradation products	UPLC-QToFMS
Chiappini (19)	Case-control Lipidomic analysis on human liver biopsies including normal liver, nonalcoholic fatty liver and NASH; adult patients. NAFL (n = 39) and NASH (n = 22); controls (n = 7).	↑6 fatty acids in NASH (C14:0, C16:0, C16:1n-7, C18:1n-7, C18:1n-9 and C18:2n-6). Eicosanoid precursors [arachidonic acid (C20:4n-6), eicosapentaenoic acid (C20:5n-3) and docosahexaenoic acid (C22:6n-3)] ↓in livers of NASH compared to controls.	UPLC-QQQ GC-MS TOF-SIMS
Scupakova (20)	Fresh frozen liver biopsies from adult obese subjects undergoing bariatric surgery with various degrees of NAFLD Fresh frozen liver biopsies from obese subjects undergoing bariatric surgery (n = 23), non-steatosis n = 7; steatosis n = 16.	Phosphatidylinositols and arachidonic acid metabolism was associated with nonsteatotic regions, whereas low density lipoprotein and very low density lipoprotein metabolism was associated with steatotic tissue.	MALDI-MSI
Combined explorations in plasma and liver tissue			
Gorden (21)	Profiling of lipids from plasma, liver biopsies,and urine samples in adult patients classified on the basis of liver histology as normal, steatotic, NASH, or cirrhotic NAFL (n = 17) and NASH (n = 20); Cirrhosis (n = 20), controls (n = 31).	The sphingolipid species that differed in both the liver and plasma across histological categories were longer chain ceramides, dihydroceramides, or 1-deoxy-dihydroceramides. Liver: differences between NASH and steatosis in TAG acyl chain composition followed a pattern of ↑amounts of short and saturated fatty acyl chain-containing species in NASH and ↓ amounts of PUFA-containing TAGs	Multiple MS platforms

GC-FID, gas-chromatography flame-ionization detector; HETE, hydroxyeicosatetraenoic acid; HPLC, high-performance liquid chromatography; H-MRS, proton magnetic resonance spectroscopy; lysoPC, lysophosphatidylcholine; MALDI-MSI, matrix-assisted laser desorption ionization-mass spectrometry imaging; NAFL, nonalcoholic fatty liver (simple steatosis); NASH, nonalcoholic steatohepatitis; QQQ, triple quadrupole mass spectrometer; TLC, thin layer chromatography; ToF, time of flight mass spectrometry; TOF-SIMS, Time-of-Flight Secondary Ion Mass Spectrometry Imaging; UPLC, Ultra-performance liquid chromatography.

Seminal studies in this field have uncovered differences in the plasma lipidomic profile of patients with NAFLD across the entire disease spectrum. For example, Puri *et al.* (8) showed that the progression from normal to fatty liver [simple steatosis (NAFL)] to NASH involves a stepwise increase in lipoxygenase metabolites 5(S)-hydroxyeicosatetraenoic acid (5-HETE), 8-HETE, and 15-HETE. Feldstein *et al.* (9) demonstrated that lipid peroxidation products, including hydroxy-octadecadienoic acids, 9-hydroxy-octadecadienoic acid and 13-hydroxy-octadecadienoic acid, oxo-octadecadienoic acids (9-oxoODE and 13-oxoODE), and products of free radical-mediated oxidation of linoleic acid, are elevated in plasma samples of patients with NASH compared with patients with hepatic steatosis and normal liver biopsy.

Findings yielded by subsequent studies have implicated the lysophosphatidylcholine species in the disease severity and have demonstrated the importance of distinctive lipid species for differentiating the histological stages (Table 1). In fact, several biomarkers (22, 23) and commercial panels (24, 25) have emerged from the combination of plasma metabolomic and lipidomic profiling.

Likewise, authors of numerous studies have profiled the lipidomic signature of NAFLD-liver tissue, which has been of remarkable value to the understanding of disease mechanisms (Table 1). This is particularly relevant as tissue lipidomics studies have been carried out despite the tremendous difficulties in obtaining liver samples from affected patients and the significant challenges that have to be overcome in sample processing and analysis.

In their pioneering study, Puri and coworkers (16) used a lipidomic approach to quantify the major lipid classes and the distribution of fatty acids in the liver of NAFLD patients to explain the potential impact of liver lipid species on the development and progression of NASH. The authors showed that the n-6 polyunsaturated fatty acid content in the total lipids was correlated with the disease extent, whereby it was the lowest in the controls followed by those diagnosed with fatty liver and finally NASH. Increased lysophosphatidylcholine levels were also detected in the liver of NASH patients (16). In addition, the authors observed a decrease in the downstream n-6 (arachidonic acid: 20:4n-6) and n-3 (eicosapentanoic acid: 20:5n-3, docosahexanoic acid: 22:6n-3) polyunsaturated fatty acids in both NAFL and NASH samples (16).

Scupakova *et al.* (20) similarly observed that phosphatidylinositols and arachidonic acid-related metabolism were associated with nonsteatotic regions. In contrast, low density lipoprotein and very low density lipoprotein metabolism was associated with steatotic tissue. The key characteristics and findings of all studies involving the characterization of lipid species in the liver tissue are shown in Table 1.

## Integration of NAFLD Lipidomics to Outline Molecular Phenotypes

### Dysregulated disease pathways inferred from lipidomics studies

Human metabolome, including the lipidome, is influenced by several factors such as genetic predisposition and the interaction with the environment. The latter involves not only diet and lifestyle but also the interaction with the whole body microbiome.

In this work, we adopted a strategy based on systems biology and network analysis derived in part from multi-OMICs experiments to understand the functional consequences of the mutual interactions between not only intrinsic factors, including genetics, but also external ones such as the microbiota, diet, and lifestyle. Integration of biological and OMICs data into pathway analysis may facilitate understanding of novel molecular signatures that would ultimately serve in biomarker and drug discovery, thus fueling future research progress. Thus, integrating several biochemical reactions composed of genes, metabolites, and proteins with clinical information can help in deciphering dysregulated disease pathways as well as identifying molecular phenotypes associated with a disease trait.

For this purpose, we text-mined lipidomics studies in NAFLD and NASH by using the PubTator Central search engine available at https://www.ncbi.nlm.nih.gov/research/pubtator, which is a web-based system providing automatic annotations of biomedical concepts in PubMed abstracts and PubMed Central full-text articles. We also employed the LipidPedia search engine available at https://lipidpedia.cmdm.tw/, which is a lipids encyclopedia of biomedical information. When searching PubTator Central, we used “lipidomics OR (metabolomics AND lipids) AND (NAFLD OR NASH) AND human∗” search string, while adopting the disease term “fatty liverMeSH ID D005234” for searching the LipidPedia.

To ensure proper name entity recognition of the retrieved metabolites, including abbreviation and synonym recognition, we manually curated the list of terms. A final list of 70 metabolites, mostly lipids, and a list of 61 genes were used to perform further pathway and enrichment analyses provided at the MetaboAnalyst—a web-based platform freely available at https://www.metaboanalyst.ca. The complete list of metabolites, including the HMDB (Human Metabolome Database) ID and PubChem number is shown in Supplemental Table 1. The list of genes associated with lipidomic studies that was used to perform metabolite-gene-disease interaction networks is shown in Supplemental Table 2.

**Fig. 1:**
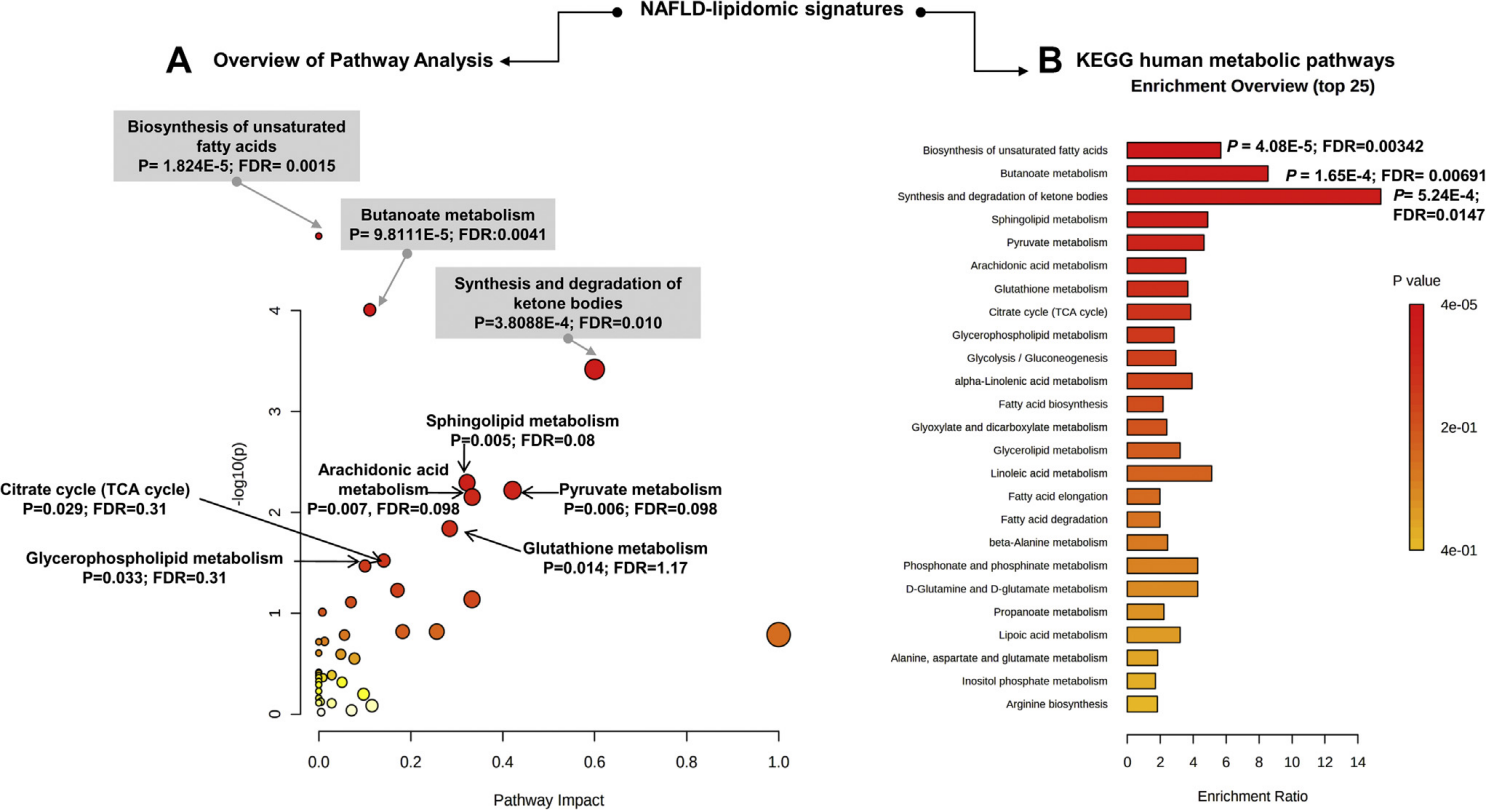
Pathway analysis derived from NAFLD-lipidomics. Pathway (A) and enrichment (B) analyses were performed by integrating the list of query metabolites (mostly lipids) obtained through data mining and retrieved from the Homo sapiens (KEGG) dataset. The following parameters were used for topology analysis (relative-between's-centrality) and Enrichment method (hypergeometric test). The complete list of metabolites used for pathway prediction is shown in Supplemental Table 1. Enrichment analysis: one-tailed *P*-values are provided after adjusting for multiple testing. FDR, false discovery rate; NAFLD, nonalcoholic fatty liver disease; KEGG, Kyoto Encyclopedia of Genes and Genomes.

Pathway (Fig. 1A) and enrichment analysis (Fig. 1B) were performed by integrating the list of query metabolites into Homo sapiens Kyoto Encyclopedia of Genes and Genomes database. Nine Kyoto Encyclopedia of Genes and Genomes significantly dysregulated pathways (*P* < 0.05) were found, including biosynthesis of unsaturated fatty acids, butanoate metabolism, and synthesis and degradation of ketone bodies that had the highest *P* values, along with sphingolipid metabolism, arachidonic acid metabolism, and pyruvate metabolism (Fig. 1A). The pathway level *P*-values were further integrated into a final ranked list of perturbed pathways based on the enrichment ratio (Fig. 1B). Because of multiple sources of evidence (7), the pathway analysis results are not only biologically plausible but are also certainly expected. Nevertheless, a dysregulated pathway was identified that not only reached high significance but also might explain novel disease mechanisms linking NAFLD susceptibility, diet, and the gut microbiome. This pathway is butanoate metabolism, and it will be discussed in detail in the subsequent section of this review.

**Fig. 2:**
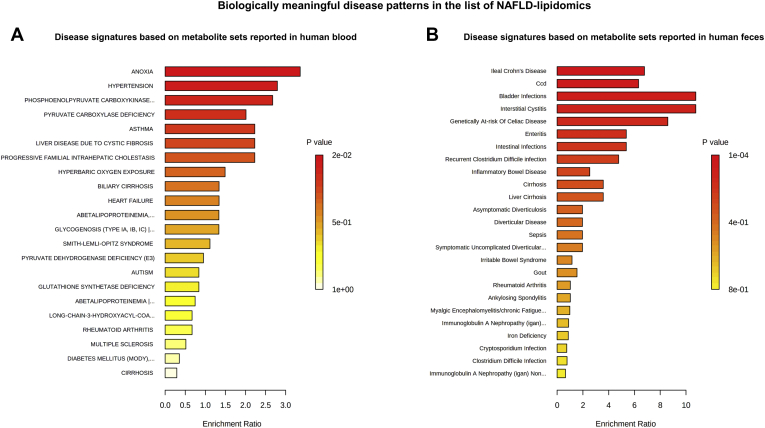
Enrichment on disease signatures based on NAFLD lipidomic studies. Overenrichment (ORA) analysis was performed by integrating the list of query metabolites (Supplemental Table 1) and disease signatures retrieved from the MetaboAnalyst platform based on metabolite sets reported in human blood (A) and human feces (B). ORA was implemented using the hypergeometric test to evaluate whether a particular metabolite set is represented more than expected by chance within the given compound list. One-tailed *P*-values are provided after adjusting for multiple testing. NAFLD, nonalcoholic fatty liver disease.

To identify biologically meaningful disease patterns in the list of NAFLD-lipidomic terms, we performed enrichment analysis based on the disease signatures retrieved from the MetaboAnalyst platform that are derived from metabolite sets reported in human blood or feces (26, 27). We found a significant overlap between the list of NAFLD-lipidomic–related metabolites and diverse disease signatures. For example, among the blood-related disease signatures, we found the highest significant scores for molecular signatures associated with anoxia and hypertension (Fig. 2A). Conversely, among the fecal-related disease signatures, we found significant scores for bowel inflammatory diseases, intestinal infections, and cirrhosis (Fig. 2B).

### Butanoate metabolism in NAFLD biology: a novel lipidome-related phenotype?

Our pathway analysis identified butanoate metabolism as one of the most dysregulated lipidome-related signatures in NAFLD and NASH. Butyrate is produced as an end-product of a fermentation process performed by obligate anaerobic bacteria. Butyrate metabolism—also known as butanoate metabolism—describes the metabolic fate of several short-chain fatty acids or short-chain alcohols that are typically produced by intestinal fermentation (28). Many of these molecules are eventually used in the production of ketone bodies, in the creation of short-chain lipids or as precursors to the citrate cycle, glycolysis, or glutamate synthesis (29). Butanoate metabolism is also associated with microbial-derived metabolites that might act locally in the liver tissue (30) or systemically (31).

The role of butyrate derived from intestinal bacterial has gained substantial attention in the last decade because of its putative biological properties in maintaining gut health. Specifically, available empirical evidence indicates that butyric acid influences colonocytes' gene expression by inhibiting histone deacetylases (32), interferes with proinflammatory signals in epithelial cells such as NF-κB (33), and downregulates proinflammatory effectors in macrophages of the lamina propria in the intestinal mucosa (34). Thus, through these mechanisms, butyric acid might prevent and inhibit colon carcinogenesis.

Nevertheless, the potential beneficial effects of butyrate on human health are still under debate.

For example, it is known that butyrate modulates intestinal permeability, allowing translocation of several bacterial taxa, including *Salmonella* (35), which is an undesirable effect. In addition, paradoxical effects of butyrate on obesity may potentially indicate its detrimental role in systemic metabolism (36). Most importantly, the role of butyric acid in NAFLD pathogenesis has not yet been established. For instance, Huang *et al.* (37) recently reported that maternal butyrate supplementation leads to insulin resistance and ectopic lipid accumulation in the skeletal muscle of the offspring. Zhou *et al.* (11) similarly demonstrated that taking maternal sodium butyrate supplement during pregnancy and in the lactation period promotes maternal fat mobilization, which may result in fatty acid uptake and lipid accumulation in the liver of the offspring. Other lines of evidence suggest that butyrate is able to increase lipid synthesis from acetyl-CoA or ketone bodies via the β-hydroxy-β-methylglutaryl-CoA pathway, which might directly promote obesity (38). Findings reported by Macfarlane *et al.* (39) also suggest that butyrate transported via the portal vein could be involved in liver lipid biosynthesis.

On the contrary, as indicated by the data from a mouse study, butyrate supplementation is shown to alleviate the severity of diet-induced NASH phenotype (40). This might suggest that, at least in a rodent model of NAFLD, butyrate reveals antisteatotic and antiinflammatory effects.

Collectively, this evidence prompts the question of whether activation of butanoate pathway in the context of human NAFLD is beneficial or detrimental.

To understand the potential clinical associations of butanoate metabolism and NAFLD, we analyzed the epidemiological data retrieved from the National Health and Nutrition Examination Surveys (NHANES) 2017–2018 database. This data set is freely available online at https://www.cdc.gov/nchs/nhanes/index.htm. The NHANES is a collection of population-based surveys conducted by the National Center for Health Statistics of the Centers for Disease Control and Prevention of the United States. The National Center for Health Statistics Research Ethics Review Board approved the NHANES protocol, and informed consent was obtained from all participants. Our analysis focused on liver steatosis, defined by the controlled attenuation parameter >268 dB/m, obtained via transient elastography (FibroScan®). In addition, for inferring butyric acid levels, daily total energy and nutrient intake from food and beverages was obtained for each participant through dietary recall surveys. Butyric acid (variable ID: DR1TS040) was obtained by extrapolating dietary intake data pertaining to 7,484 individuals.

In linear regression analysis, NAFLD was associated with high butyric acid intake (mean ± SD: 0.77 ± 0.50 g; low intake: 0.15 ± 11 g) independently of gender, waist circumference, and type 2 diabetes or glycohemoglobin (OR: 1.21, 95% CI: 1.02–0.43, *P* = 0.035). We thus modeled the relationship between NAFLD and butyric acid by linear logistic regression with an interaction term for butyric acid intake and waist circumference while adjusting for subjects' demographic and clinical characteristics. We used waist circumference as main covariate because we previously found that excess of abdominal adipose tissue is a common risk factor for NAFLD not only in obese but also in lean subjects (41).

**Fig. 3:**
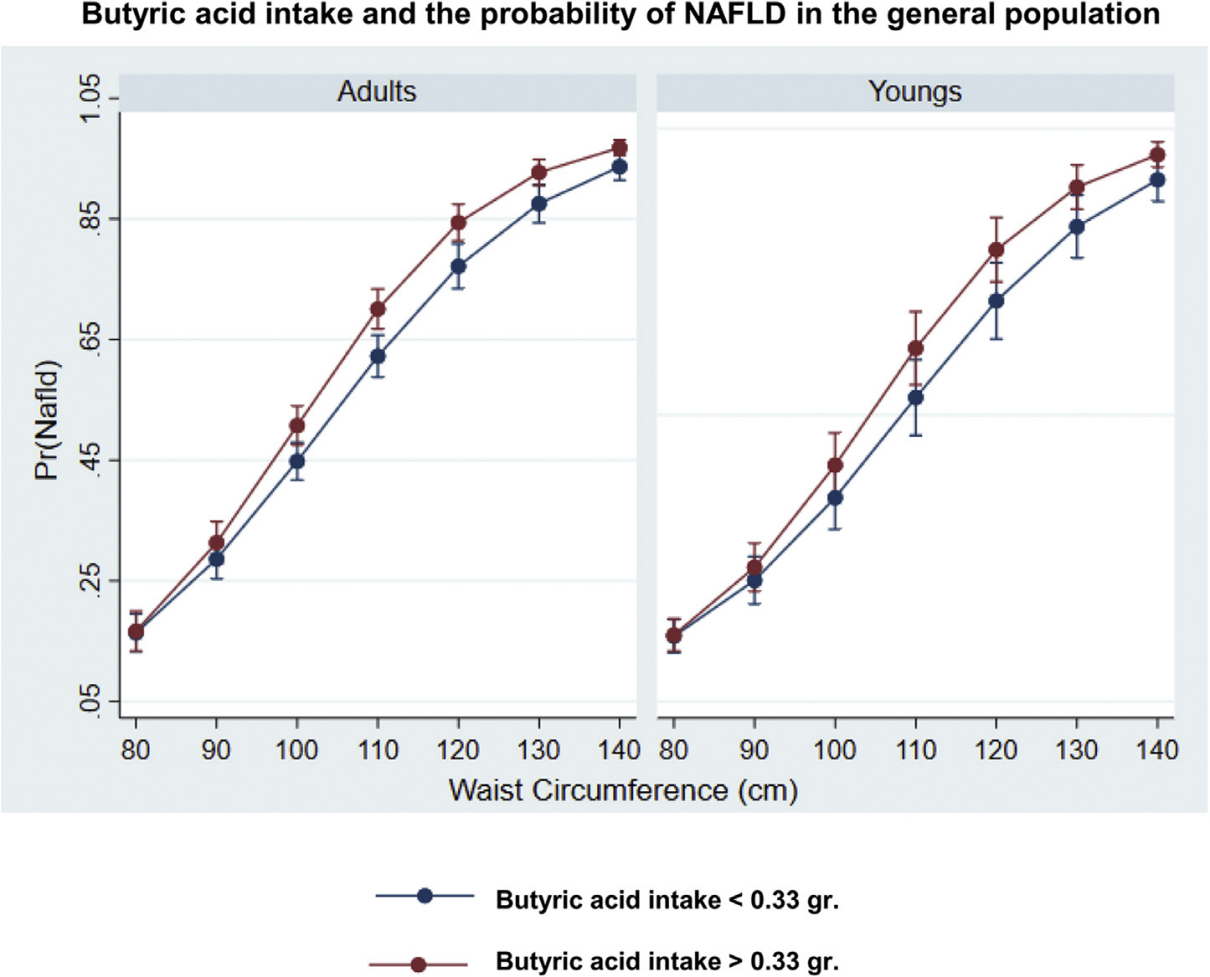
The relationship between NAFLD and butyric acid: Analysis of data yielded by the National Health and Nutrition Examination Surveys 2017–2018. We used the data pertaining to fatty acid intake, such as saturated fatty acids (SFAs) 4:0 (butanoic), reported as a part of the 24-h diet recall (https://wwwn.cdc.gov/Nchs/Nhanes/2017-2018/DR1TOT_J.htm#DR1TS040). Cut-off threshold for butyric acid was based on the median level (butyric acid intake </> 0.33 g/day) based on the reports of 7,484 participants. As described in the main text, 1,876 of these subjects were classified as having non-NAFLD and 1,483 as having NAFLD. The relationship between NAFLD and butyric acid was modeled using the STATA v16 package by linear logistic regression with an interaction term for butyric acid intake and waist circumference while adjusting for subjects' demographic and clinical characteristics. The Margins routine was used for estimating the probability of having NAFLD in each stratum, as well as for determining statistical significance and visualization of key findings. Plots show the interaction between butyric acid intake and waist circumference after adjusting for confounding factors such as age, total energy, and cholesterol consumption and categorical variables such as gender, diabetes, and ethnicity. NAFLD risk increased steadily with waist circumference but was significantly higher in subjects ingesting butyric acid above the median level for the population, except in those with waist circumference below ∼100 cm. Pr (NAFLD): probability of having NAFLD. NAFLD, nonalcoholic fatty liver disease.

Figure 3 shows the interaction between butyric acid intake and waist circumference after adjusting for confounding factors such as age, total energy, and cholesterol consumption, and categorical variables such as gender, diabetes, and ethnicity. Although NAFLD risk increased steadily with waist circumference, it was significantly higher in subjects ingesting butyric acid above the median level, except in those with waist circumference below ∼100 cm.

There is scarce clinical evidence on the putative association between butyric acid and human NAFLD. Nevertheless, recent epidemiological studies demonstrated potential detrimental effects of butyric acid on human metabolic health. For instance, explorations of the relationship between fatty acids (FAs) and frailty and mortality risk conducted as a part of the NHANES population study showed that after adjusting for potential covariates and the higher total FAs, saturated FAs and butanoic acid intake were associated with a higher degree of frailty not only among older but also among middle-aged individuals (42). This finding is relevant as frailty, which is characterized by a decline in functioning across multiple organ systems, accompanied by increased vulnerability to stressors, is an emerging global health burden with significant implications for clinical practice and public health (43).

Together, these data suggest that a metabolite imbalance in the butanoate metabolism pathway may play a significant role in NAFLD pathogenesis and might have adverse systemic consequences that differ from the local protective effects against intestinal injury.

Tributyrin—also known as 1,2,3-tributyrylglycerol, glycerol tributyrate, or glyceryl tributyrate—is a triglyceride obtained by formal acylation of the three hydroxy groups of glycerol by butyric acid. Tributyrin is naturally present in butter and is used as an ingredient in making margarine. Most importantly, tributyrin must undergo chemical conversion by metabolic processes before becoming the pharmacologically active substance for which it is a prodrug.

**Supplemental Figure S1:**
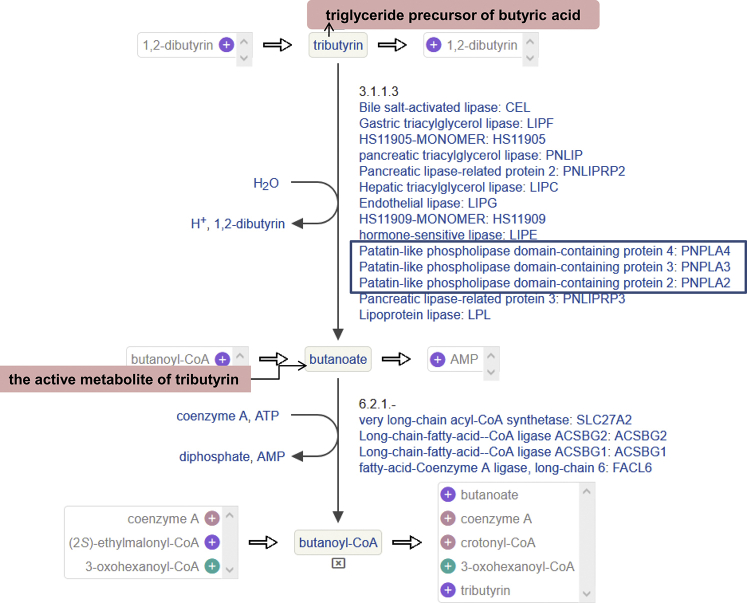
Butyrin (BioCyc Id CPD-13014) metabolism and the role of PNPLA3 enzyme.

Interestingly, interactive exploration of the metabolic network around tributyrin using the HumanCyc—an encyclopedic reference on human metabolic pathways—showed that some key NAFLD-related enzymes, including PNPLA2, PNPLA3, and PNPLA4 participate in the conversion pathway of tributyrin (BioCyc Id CPD-13014) to butanoate (Supplemental Fig. 1). Still, it remains to be explored whether genetic variation in *PNPLA3*, particularly rs738409 that is the major genetic modifier of NAFLD (44) and the disease severity (45, 46), has a differential role in the conversion of tributyrin into butanoate. Likewise, it is unknown whether any impairment associated with PNPLA3 protein function because of the amino acid change (p.Ile148Met) of the missense rs738409 variant might eventually mediate the putative detrimental effects of butanoate on NAFLD pathogenesis.

## From Perturbed Lipid Homeostasis to Biomarker and Drug Target Prediction

The text-mined metabolite list linked to the NAFLD lipidome was used to perform enrichment analyses based on chemical structures and drug pathways. These analyses were based on several libraries assembled in the MetaboAnalyst platform containing ∼9,000 biologically meaningful metabolites primarily identified through human studies and including >1,500 chemical classes (26, 27).

**Fig. 4:**
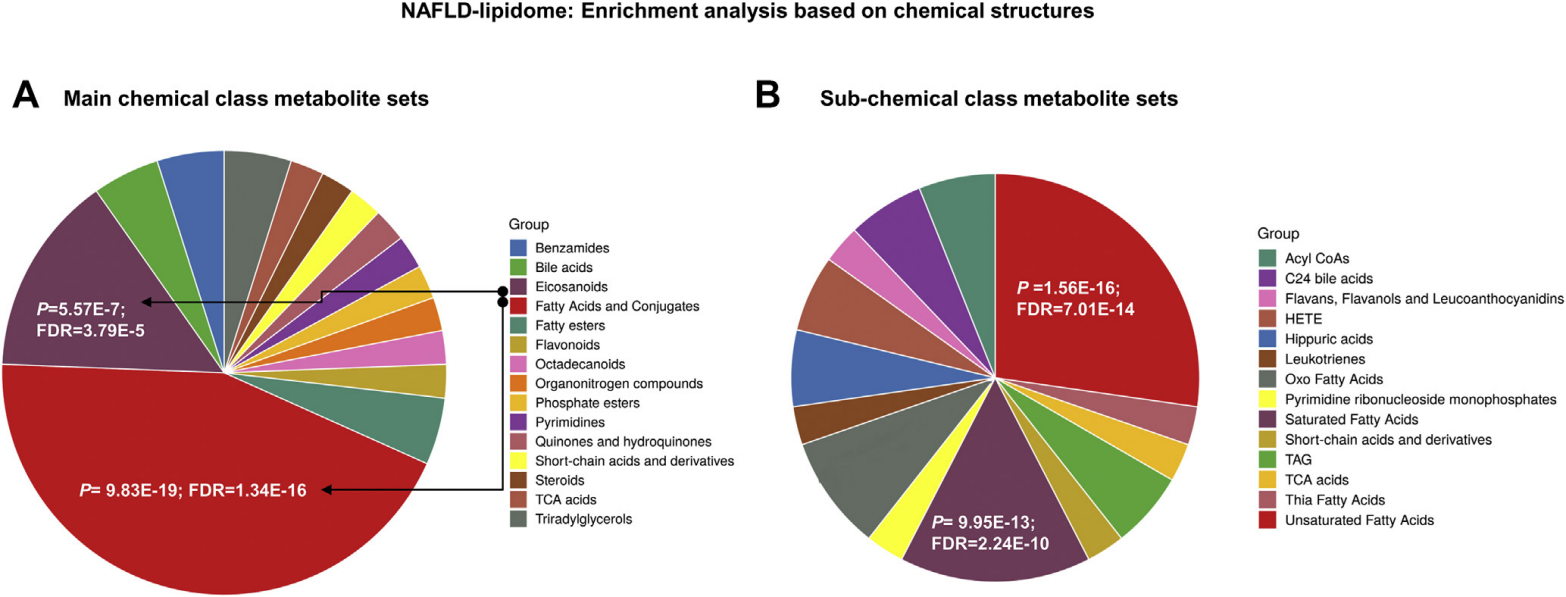
Enrichment analysis based on chemical structures. Enrichment analysis (ORA) was performed by integrating the list of query metabolites (shown in Supplemental Table 1) and a set of main chemical (A) and subchemical (B) class metabolites gathered in the MetaboAnalyst platform. ORA was implemented using the hypergeometric test to evaluate whether a particular metabolite set is represented more than expected by chance within the given compound list. FDR, false discovery rate; NAFLD, nonalcoholic fatty liver disease.

The enrichment analysis based on chemical structures revealed high representation of fatty acids and conjugates, eicosanoids, and short-chain acids and derivatives (Fig. 4A), whereas enrichment based on subchemical class metabolite sets or lipid sets (Fig. 4B) showed high representation of unsaturated and saturated fatty acids, HETE,OXO-fatty acids, and C24 bile acids that constitute a major part of the bile and which may also be deconjugated by bile salt hydrolases of some bacterial genera of the gut microbiota (47).

**Fig. 5:**
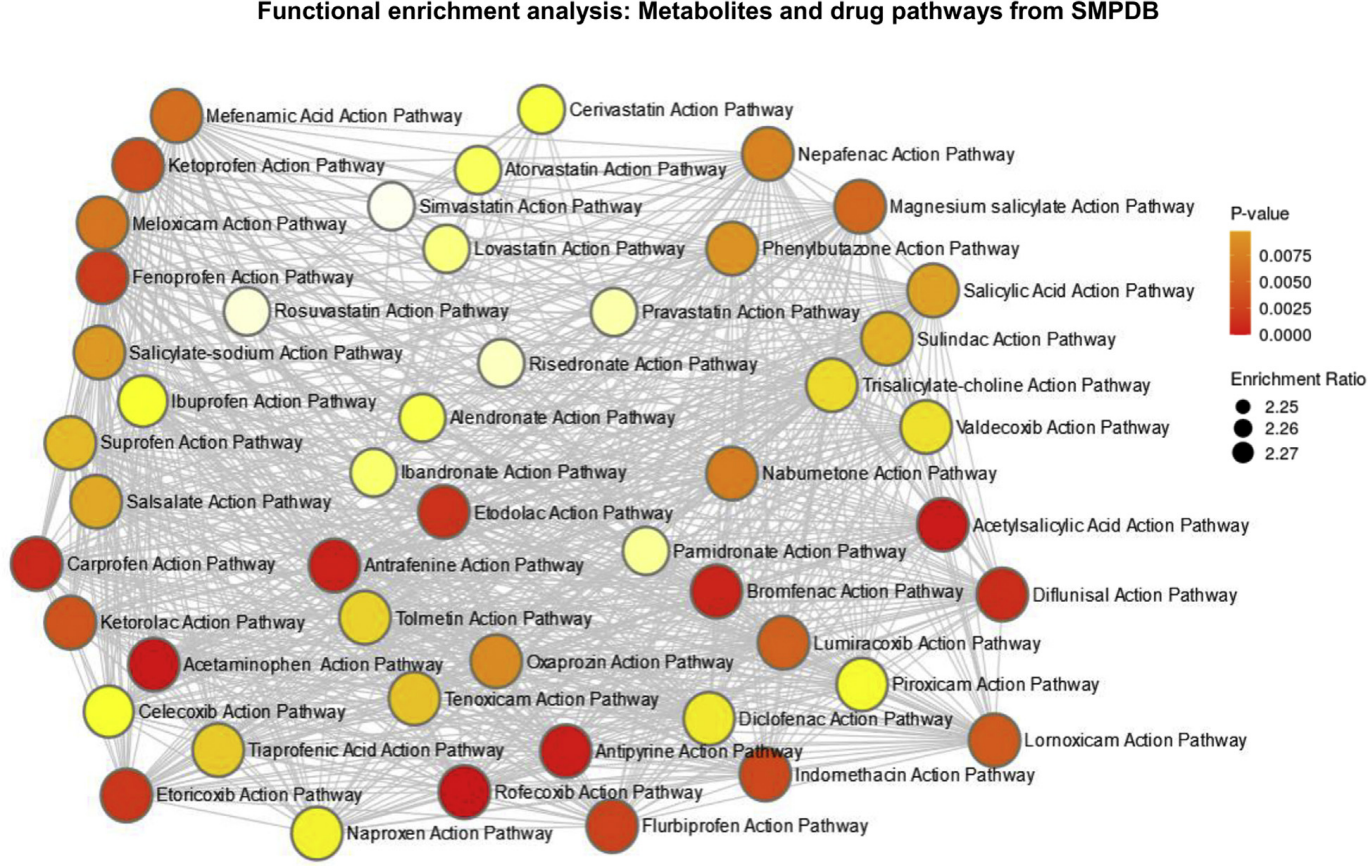
Enrichment analysis based on drug pathways from SMPDB. Enrichment analysis (ORA) was performed by integrating the list of query metabolites (shown in Supplemental Table 1) and drug pathways from The Small Molecule Pathway Database (SMPDB). ORA was implemented using the hypergeometric test to evaluate whether a particular metabolite set is represented more than expected by chance within the given compound list. One-tailed *P*-values are provided after adjusting for multiple testing.

Analyses focusing specifically on drug pathways from The Small Molecule Pathway Database showed enrichment on rofecoxib, acetaminophen, acetylsalicylic, antipyrine, and antrafenine action pathways, among many other related nonsteroidal antiinflammatory drug pathways (Fig. 5). These findings are not only biologically plausible but are also highly anticipated because of the redundancy of arachidonic acid, leukotrienes, and prostaglandins in the enrichment analysis.

## Integrated Pathway-Level Analysis of Genes and Lipidomic Signatures Associated With NAFLD

### Joint analysis of genomic and lipidomic data derived from NAFLD studies

Genome-wide association studies with high-throughput metabolic profiling showed high heritability among components of the human metabolome (48).

To exploit the application of biomedical text mining on NAFLD biology and to integrate existing knowledge into biological networks, we performed joint pathway analysis of NAFLD genetic and lipidomic data. For this integrated analysis, we used the IMPaLA (Integrated Molecular Pathway Level Analysis) resource freely available at http://impala.molgen.mpg.de/, which allows combining data sets and analyzing both types of data simultaneously (49). Pathway overrepresentation with joint genetic and lipid data were based on a comprehensive list of 1,160 loci associated with NAFLD/NASH obtained by biomedical text mining and the list of 70 metabolites/lipid species obtained through text mining, as explained in the preceding sections. Briefly, genes/proteins associated with NAFLD/NASH were searched using the Génie algorithm (50) and web server, a data mining tool available at http://cbdm-01.zdv.uni-mainz.de/∼jfontain/cms/?page_id=6. The Génie algorithm prioritizes all genes from a species according to their relation to a biomedical topic using all available scientific abstracts and orthology information (50).When searching literature, focus was given to articles and abstracts published before January 28, 2021 available in the MEDLINE, NCBI Gene, and HomoloGene databases. The training set consisted of 924 abstracts from PubMed retrieved using the query “non-alcoholic fatty liver disease NASH NAFLD non-alcoholic steatohepatitis.” Only the test set of genes related to Homo sapiens was used for our analysis (taxonomic identifier = 9606), and false discovery rate < 0.01 was used for genes.

The list of dysregulated pathways derived from the integrated gene and lipid analysis is provided in Table 2, including adjusted *P*-value (Q-value) for genes and metabolites. The analysis was narrowed to including at least 10 genes and 10 shared metabolites from the input lists in the enrichment sets and was restricted to pathways showing both Q-values ≤0.05. The top five pathways are based on the overrepresentation of genes and lipids/metabolites involved in signal transduction, selenium micronutrient network, metabolism of lipids, Class A/1 (Rhodopsin-like receptors), GPCR (G protein-coupled receptors) ligand binding, and GPCR downstream signaling, among other relevant pathways. GPCRs involve ∼103 druggable targets for the treatment of many common diseases (https://www.proteinatlas.org) and represent ∼34% of the marketed drugs (51). Notably, butyric acid is consistently involved in a large proportion of the top-ranked dysregulated pathways.

**Table 2:** Integrated pathway level analysis of genes and lipid-related metabolites associated with NAFLD from mining biological data

Pathway Source^a^	*Q* Genes^b^	*Q* Metabolites^b^
Signal transduction	3.52E-13	2.78E-27
Metabolism of lipids	1.18E-15	2.57E-19
Selenium micronutrient network	7.34E-21	8.86E-13
Class A/1 (Rhodopsin-like receptors)	2.58E-10	1.77E-20
Metabolism	2.99E-11	6.56E-16
GPCR ligand binding	4.04E-09	2.86E-16
GPCR downstream signaling	0.00898	8.48E-23
Signaling by GPCR	0.0102	1.35E-22
Fatty acid metabolism	2.18E-10	1.93E-12
G alpha (q) signaling events	0.00216	2.37E-17
Transport of small molecules	8.02E-07	8.25E-13
G alpha (i) signaling events	1.71E-07	2.42E-10
Metabolism of vitamins and cofactors	6.43E-06	1.39E-08
Phase I—Functionalization of compounds	0.000352	2.77E-06
Biological oxidations	0.000708	7.40E-06

a Pathways are from REACTOME pathways except for Selenium Micronutrient Network that is from WikiPathways.

b *Q value:* the hypergeometric distribution is used to assess the significance of each pathway in terms of its overlap with those lists; *Q*-values are calculated with the false discovery rate method.

**Fig. 6:**
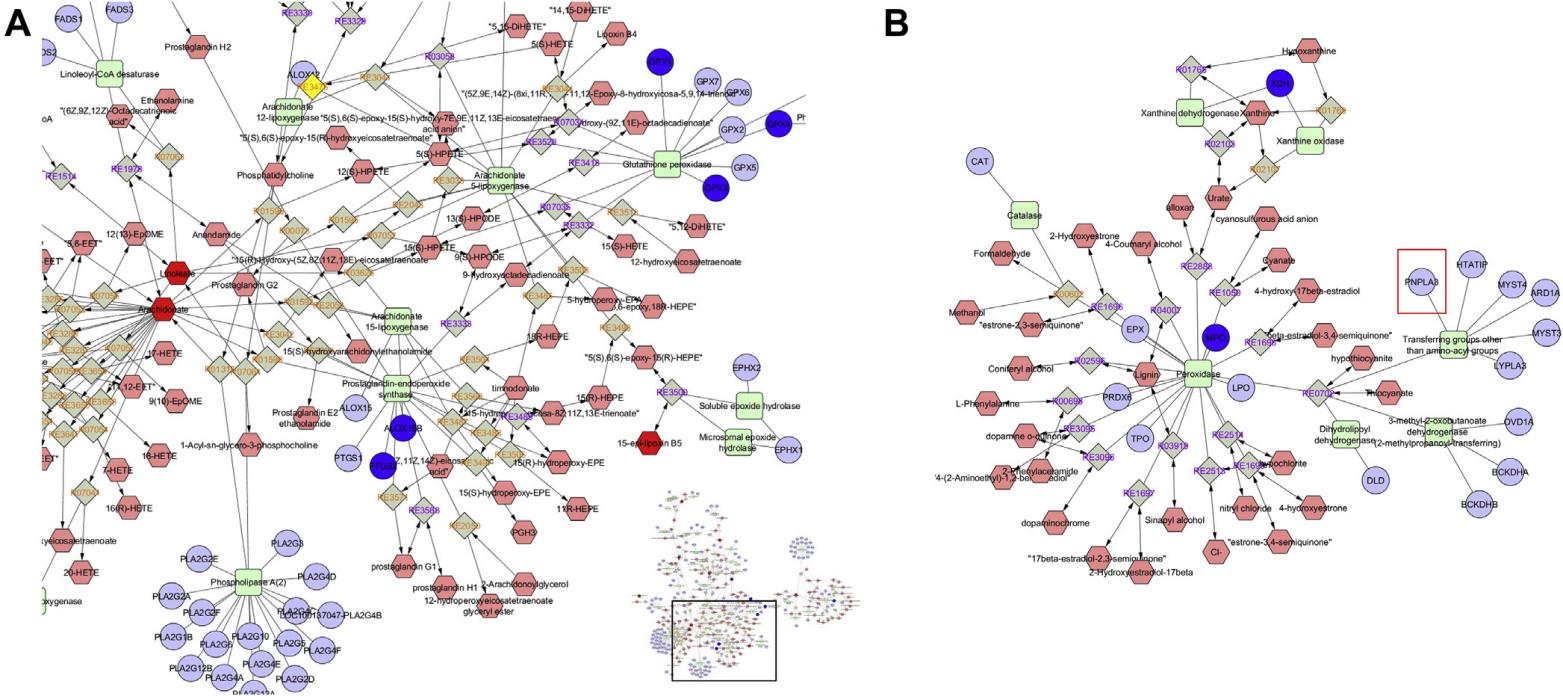
Interactome analysis based on the Selenium Micronutrient Network. Figures show the interactome of compounds, chemical reactions, enzymes, and genes associated with the derived NAFLD-lipidomics list. Compounds are denoted by *pink hexagons*, genes by *blue circles*, enzymes by *green squares*, and reactions by *gray squares* with purple or orange label text to represent reversible and nonreversible reactions, respectively. Selected nodes are highlighted in yellow. The interactome was built using MetsSape (52), a plugin for the widely used network analysis software Cytoscape that supports calculation, analysis, and visualization of gene-to-metabolite networks in the context of metabolism. The presentation tier consists of the plugin for Cytoscape. A: Inset with focus on prostaglandins, arachidonate, linoleic acid, and HETEs. B: Node associated with peroxidases and transference of groups from amino-acyl-groups, focusing on the participation of PNPLA3. HETE, hydroxyeicosatetraenoic acid.

As an example, MetScape analysis based on the selenium-centered micronutrient biological network is shown in Fig. 6; the interaction network displays the most relevant biochemical processes related to oxidation and lipid-derived proinflammatory mediators. Overlapping metabolites (NADP, arachidonic acid, cholesterol, NADPH, thromboxane B2, alpha-Linolenic acid, leukotriene B4, (R)-lipoic acid, linoleic acid, 8-isoprostane, glutathione, eicosapentaenoicacid, and prostaglandin E2) with NAFLD genes (*MPO*, *IFNG*, *CBS*, *SELENOP*, *SAA1*, *SAA2*, *ABCA1*, *SELENOS*, *MTHFR*, *INS*, *SCARB1*, *NFKB1*, *ICAM1*, *INSR*, *GPX1*, *SERPINE1*, *GPX4*, *XDH*, *CCL2*, *APOA1*, *APOB*, *TNF*, *ALB*, *TXNRD2*, *RELA*, *HBA1*, *IL1B*, *F2*, *F7*, *LDLR*, *PTGS2*, *ALOX5*, *IL6*, *SAA4*, *GPX3*, *GGT1*, *SOD1*, *SOD2*, *CRP*, *ALOX15 B*) that joined at the main node of the network are shown in Fig. 6A. Another relevant subnetwork is shown in Fig. 6B, which includes MPO (myeloperoxidase), reactions associated with peroxidases, and genes involved in these pathways, including *PNPLA3* (Fig. 6B, inset).

Substantial selenium participation in the modulation of metabolism was also noted. The role of selenium in the dietary management of chronic metabolic diseases has been recently summarized (53). Nevertheless, the effects of selenium supplementation on NASH progression have been poorly explored. Pathway overrepresentation with joint genetic and lipid data could lead to novel potential therapies for treating NASH. Likewise, joint genetic and lipid data analysis may aid in personalizing nutrition according to the patients' genetic makeup and needs.

### Metabolite-gene-phenotype interaction network suggests targets and disease signatures

Previous work based on Covariates for Multiphenotype Studies approach has demonstrated that genetics of lipid metabolites is strongly interconnected, harboring core regulator genes with strong pleiotropic effects (54).

**Fig. 7:**
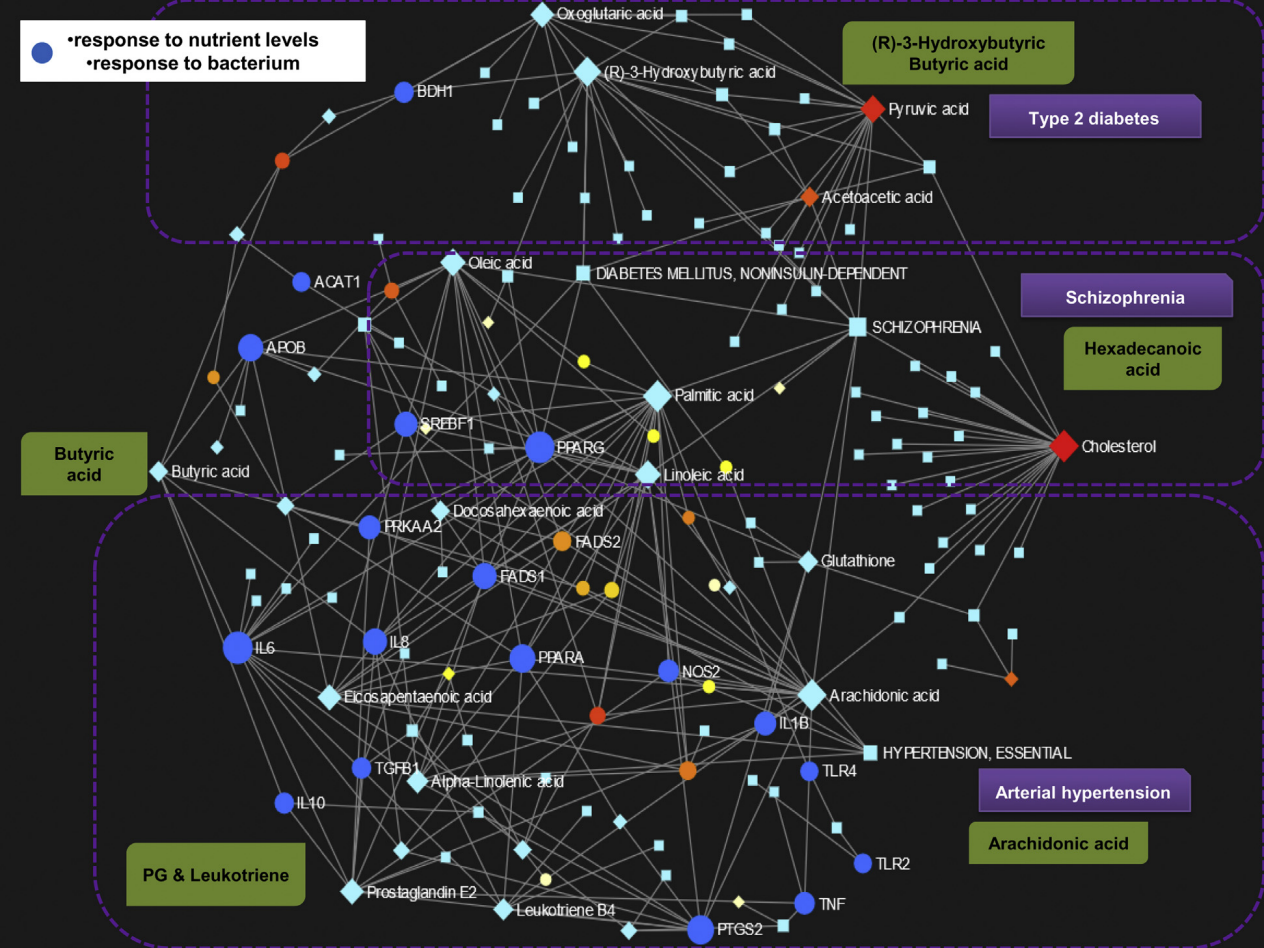
Network representation of metabolite-gene-disease interaction. The metabolite-gene-disease interaction network was generated using the Network Explorer function available through the MetaboAnalyst platform. The network was created by integrating the list of query metabolites (shown in Supplemental Table 1) and the list of genes (shown in Supplemental Table 2) obtained by data mining into PubTator Central under the term “lipidomics of NAFLD.” To generate knowledge-based networks, the input metabolites and genes (seeds) were mapped to the selected interaction network to create subnetworks containing these seeds and their direct neighbors (i.e., first-order subnetworks). This procedure yielded one large subnetwork (“continent”) with several smaller ones (“islands”). The main network integrates gene-metabolite, metabolite-disease, and gene-disease interaction networks. Each node represents either a gene (*circles*), a metabolite (*light blue diamonds*), or a disease (*light blue squares*). *Blue circles* in the figure highlight genes and their related metabolites involved in response to nutrient levels and bacterium GO biological pathways. Each edge indicates an association between one gene and one metabolite or one disease. *Circle*, *diamond*, or *square* size is directly proportional to the number of other nodes associated with it. A filter on “response to nutrient levels” and “response to bacterium” was used to create the network depicted in the figure.

Hence, we created a metabolite-gene-phenotype interaction network by integrating the list of 70 metabolites and 61 genes generated by mining lipidomic studies on NAFLD from the PubTator Central, as outlined in previous sections. This analysis allows the identification of connections that cross pathway boundaries (e.g., metabolite-disease interactions) and provides a global view of the potential functional relationships among metabolites, connected genes, and target diseases. The network is indeed an integration of gene-metabolite, metabolite-disease, and gene-disease interaction networks. The analyses revealed 120 enriched pathways (Gene Ontology—GO biological processes), whereby Fig. 7 shows the plot of the created interaction network. However, we specifically focused on two of the top overrepresented nodes—response to nutrient levels and response to bacterium. These two significantly dysregulated pathways—response to nutrient levels (*P* = 1.16E-12; FDR: 1.05E-10) and response to bacterium (*P* = 5.36E-10; FDR: 2.09E-08)—emphasize the relevance of environmental factors in the biology of NAFLD and its interrelationship with the lipidome, both of which are plausible intervention targets.

Furthermore, the most dysregulated pathways were also associated with regulation of lipid metabolic process, lipid biosynthetic process, and fatty acid metabolic processes. In addition, the network analysis revealed three distinctive disease signatures, namely diabetes mellitus, schizophrenia, and arterial hypertension. Diabetes (noninsulin dependent) was highly connected with the (R)-3-hydroxybutyric acid node, as well as oxoglutaric and pyruvic acid; schizophrenia with oleic and palmitic acid nodes, as well as with acetoacetic acid; and arterial hypertension with arachidonic, docosahexaenoic, eicosapentanoic, and linoleic acid pathways. Figure 7 also highlights the importance of butanoate metabolism and its interrelationship with inflammatory signals, including cytokine response (*IL6* and *IL-8*), *APOB* (apolipoprotein B), and *PRKAA2* (Protein Kinase AMP-Activated Catalytic Subunit Alpha 2)—an important energy-sensing enzyme that monitors cellular energy status, which is activated in response to cellular metabolic stresses and is a metformin's target (55). Peroxisome proliferator-activated receptor γ and α show numerous associations with several genes and metabolites of the network (Fig. 7), which reinforces the importance of focusing on targets in this subfamily of nuclear receptors for treating NASH (2).

## Druggable Targets Associated With NAFLD

**Fig. 8:**
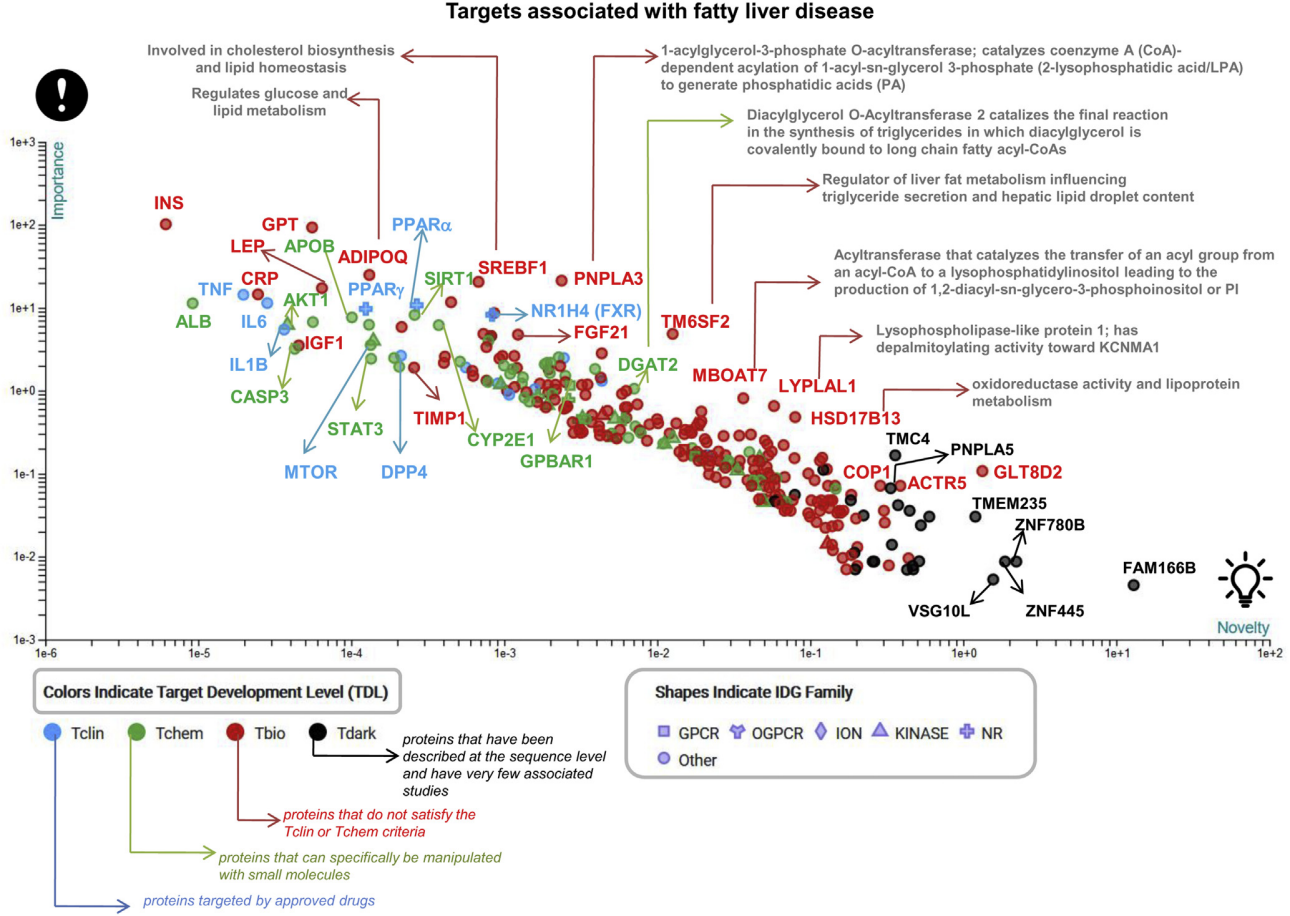
Targets associated with fatty liver disease: importance & novelty explorer. The plot depicts targets associated with NAFLD with the emphasis on proteins linked to lipid metabolism. Targets are plotted with log-log importance–novelty axes according to the algorithm implemented by the TIN-X resource (56). Briefly, the approach is guided by the following assumptions: the target that is mentioned in many abstracts that also note a specific disease is likely to be of importance to that disease; a target or disease that is mentioned in fewer abstracts is more novel and less well understood; abstracts which mention only a few targets and diseases are more specific and should be given greater weight than those in which many targets and diseases are featured (56). Targets with stronger associations and likely a greater number of publications are located in the upper-left part of the plot. Targets are classified according to the Target Development Level (https://newdrugtargets.org/): Tclin, T, Tchem, Tbio, and Tdark as explained in the figure.NR, nuclear receptor; IDG, Illuminating the Druggable Genome (IDG) Program.

The druggable-NAFLD genome/proteome is overrepresented by genes and proteins linked to lipid metabolism. Figure 8 depicts the identified NAFLD-related targets classified by biological importance and novelty. Ranking and prioritization of targets, including known modifiers of NAFLD initially discovered by genome-wide association studies and primarily replicated in NAFLD patient cohorts confirmed by liver biopsy, such as *PNPLA3* (44, 45), *TM6SF2* (12, 57, 58), and *HSD17B13* (59, 60, 61), among many other targets, were performed by the TIN-X (Target Importance and Novelty eXplorer) available at https://newdrugtargets.org/. TIN-X is a web-based tool that explores the relationships between proteins and diseases extracted from scientific literature (56). By harmonizing and integrating various sources of information, the plot presented in Fig. 8 illustrates not only targets that are currently under investigation for the treatment of NASH (2), e.g., nuclear receptors (PPARγ and α, and NR1H4), FGF21, MTOR, CASP3, DPP4, IL6, or STAT3—that is characterized by a high druggability index (5)—but also potentially novel drug targets the function and/or use of which either in NASH or in other human diseases has never been explored. These novel predicted targets based on big data analysis and systems-level models for diseases and drug action are classified according to protein and domain family, including GPCRs, ion channels, and kinases. For instance, G protein-coupled bile acid receptor 1 (also known as TGR5) emerged as a novel potential target that may also act as an epigenetic modifier. This gene encodes a member of the GPCR superfamily. The G protein-coupled bile acid receptor 1 enzyme functions as a cell surface receptor for bile acids and is implicated in the suppression of macrophage functions and regulation of energy homeostasis by bile acids.

Interestingly, proteins predicted by the Tdark resource (Fig. 8), including Zinc finger proteins (ZNF780 B or ZNF445), FAM166 B, VSG10 L, and TMEM235, are targets about which virtually nothing is known. In fact, Tdark-predicted proteins do not have known drug or small molecule binding activities/domains. Leveraging this data to investigate molecular targets for the treatment of NASH may expand the spectrum of drug candidates and/or may be used as a framework for drug repurposing (62).

## Concluding Remarks

Integration of new knowledge through systems biology approach, including large-scale OMICs data, is a robust and useful strategy to explore the molecular signatures associated with NAFLD disease severity. Pathway analysis highlighted butanoate metabolism as one of the most dysregulated lipidome-related signatures in NAFLD and NASH. Hence, while further research on this topic is certainly needed, it appears that butyric acid may be regarded as a novel lipidome-related NAFLD phenotype that might explain disease mechanisms linking NAFLD susceptibility, diet, and the gut microbiome. Nevertheless, butyric acid might exhibit differential local effects in the intestine and the liver that could be either beneficial or detrimental. Additional evidence is therefore required to disentangle the putative positive and negative impact of butanoate metabolism, including its participation in translocation of intestinal bacteria to the liver, the putative involvement in liver fat accumulation, and NAFLD-fetal programming. Drug pathways prediction and integrated pathway-level analysis of genes and lipid-related metabolites highlighted nonsteroidal antiinflammatory drug pathways and selenium micronutrient network, respectively. Integration of gene-metabolite, metabolite-disease, and gene-disease interaction networks with a specific focus on nutrition and the microbiome suggests that NAFLD, type 2 diabetes, schizophrenia, and arterial hypertension are highly interconnected by molecular signatures.

However, potential bias in the data extracted through data mining suggests that further research is needed to confirm and validate the molecular signatures and pathway predictions yielded by the present work. Despite these limitations, the findings presented here strongly suggest that novel and unanticipated associations derived from system biology have the potential to open new research opportunities, including biomarker and drug discovery.

## Supplemental data

This article contains supplemental data.

## Conflict of interest

The authors declare that they have no conflicts of interest with the contents of this article.
