# Supplementary material for: The lipidome in nonalcoholic fatty liver disease: actionable targets
Source: J Lipid Res. 2021 May 5;62:100073. doi: 10.1016/j.jlr.2021.100073 (PMC8121699; doi:10.1016/j.jlr.2021.100073)
Supplement: Supplemental Table S1 [file mmc1.docx]

**Supplementary Table 1: Curated list of metabolites that were used to perform pathway and enrichment analyses**

| **Metabolite** | **HMDB** | **PubChem** | **KEGG** |
| --- | --- | --- | --- |
| Acetic acid | HMDB0000277 | 5283560 | C06124 |
| Butyric acid | HMDB0000268 | 9863245 | C05476 |
| Capric acid | HMDB0001852 | 444795 | C00777 |
| Stearic acid | HMDB0003733 | 16061135 | NA |
| Arachidonic acid | HMDB0004888 | 22833614 | C06135 |
| Elaidic acid | HMDB0002152 | 5280490 | C02110 |
| Linoleic acid | HMDB0002396 | 151503 | NA |
| Alpha-Linolenic acid | HMDB0005962 | 134506 | NA |
| Stearidonic acid | HMDB0008504 | 52923365 | C00157 |
| Docosahexaenoic acid | HMDB0007169 | 9543767 | NA |
| Eicosapentaenoic acid | HMDB0007034 | 53477969 | NA |
| (R)-3-Hydroxybutyric acid | HMDB0000032 | 439423 | C01164 |
| Pyruvic acid | HMDB0000554 | 15818 | NA |
| L-Ornithine monochlorohydrate/ornithine | HMDB0048630 | 131759452 | NA |
| (R)-lipoic acid | HMDB0006281 | 440985 | C06341 |
| 11b-PGF2a | HMDB0014355 | 60164 | NA |
| 8-Isoprostane | HMDB0007861 | 24779559 | C00416 |
| Leukotriene B4 | HMDB0004921 | 20057331 | C06133 |
| Thromboxane B2 | HMDB0007517 | 53478303 | NA |
| Malonyl-CoA | HMDB0005045 | 5283145 | C00861 |
| Anandamide | HMDB0007748 | 53478501 | NA |
| Cholesterol | HMDB0000331 | 44263376 | NA |
| DG(14:0/14:0/0:0) | HMDB0008759 | 53479437 | C00157 |
| TG(10:0/10:0/10:0) | HMDB0002023 | 152459 | NA |
| Cytidine monophosphate | HMDB0000363 | 91451 | C05138 |
| O-Phosphoethanolamine | HMDB0000527 | 22833529 | NA |
| LysoPC(0:0/18:0) | HMDB0031121 | 15607876 | NA |
| Palmitoleic acid | HMDB0007508 | 53478294 | NA |
| Oleic acid | HMDB0000501 | 53477696 | NA |
| Palmitic acid | HMDB0000523 | 21252251 | NA |
| Oxoglutaric acid | HMDB0000502 | 5283956 | NA |
| 3-Hydroxybutyric acid | HMDB0000893 | 10457 | C08278 |
| Acetoacetic acid | HMDB0000321 | 193530 | C02360 |
| Palmitic acid | HMDB0000523 | 21252251 | NA |
| Ceramide (d18:1/12:0) | HMDB0008009 | 24778769 | C00157 |
| 24,25-Dihydroxyvitamin D | HMDB0001220 | 5280360 | C00584 |
| 1-Phosphatidyl-D-myo-inositol | HMDB0008715 | 53479393 | C00157 |
| Fatty acid | HMDB0049059 | 131759871 | NA |
| Prostaglandin E2 | HMDB0005369 | 9544069 | C00422 |
| Deoxycholic acid | HMDB0002277 | 53477747 | NA |
| Prostaglandin E2 | HMDB0005369 | 9544069 | C00422 |
| Glutathione | HMDB0050864 | 131761595 | NA |
| Adrenic acid | HMDB0007203 | 53478078 | NA |
| O-Phosphoethanolamine | HMDB0000527 | 22833529 | NA |
| Glycerophosphocholine | HMDB0000354 | 160471 | NA |
| Phosphocreatine | HMDB0006514 | 52922050 | NA |
| NADP | HMDB0000521 | 5312400 | NA |
| NADPH | HMDB0000524 | 167758 | NA |
| Cholesterol | HMDB0000331 | 44263376 | NA |
| Cholic acid | HMDB0002261 | 1563 | NA |
| TG(10:0/10:0/10:0) | HMDB0002023 | 152459 | NA |
| TG(16:1(9Z)/16:1(9Z)/16:1(9Z)) | HMDB0008191 | 53478821 | C00157 |
| Lc4Cer | HMDB0050278 | 131761030 | NA |
| Glucosylceramide | HMDB0000402 | 5280523 | C02504 |
| Eicosapentaenoic acid | HMDB0007034 | 53477969 | NA |
| Arachidonic acid | HMDB0004888 | 22833614 | C06135 |
| SM(d18:1/18:0) | HMDB0005461 | 25240373 | C00422 |
| Coenzyme Q10 | HMDB0004914 | 20057324 | C06133 |
| DG(14:0/14:0/0:0) | HMDB0008759 | 53479437 | C00157 |
| Ceramide (d18:1/12:0) | HMDB0008009 | 24778769 | C00157 |
| Catechin | HMDB0007401 | 9543829 | NA |
| Acetyl-CoA | HMDB0005359 | 9543986 | C00422 |
| Benzo[a]pyrene | HMDB0051155 | 131761886 | NA |
| TG(10:0/10:0/10:0) | HMDB0002023 | 152459 | NA |
| Linoleic acid | HMDB0002396 | 151503 | NA |
| 5-HETE | HMDB0031127 | 53656633 | NA |
| 12-HETE | HMDB0008352 | 53478927 | C00157 |
| Alpha-dimorphecolic acid | HMDB0007867 | 52922210 | C00157 |
| 5-KETE | HMDB0014565 | 5833 | C07310 |
| Phosphorylcholine | HMDB0006734 | 53477895 | C01290 |
