# Supplementary material for: The lipidome in nonalcoholic fatty liver disease: actionable targets
Source: J Lipid Res. 2021 May 5;62:100073. doi: 10.1016/j.jlr.2021.100073 (PMC8121699; doi:10.1016/j.jlr.2021.100073)
Supplement: Supplemental Table S2 [file mmc2.doc]

**Supplementary Table 2: Curated list of gens that were used to perform metabolite-gene-disease interaction network**

| **Gene symbol** | **Gene name** |
| --- | --- |
| ACACA | acetyl-CoA carboxylase alpha |
| ACACB | acetyl-CoA carboxylase beta |
| ACAT1 | acetyl-CoA acetyltransferase 1 |
| ACOT13 | acyl-CoA thioesterase 13 |
| ACSS2 | acyl-CoA synthetase short chain family member 2 |
| ADIPOQ | "adiponectin, C1Q and collagen domain containing" |
| AGPAT1 | 1-acylglycerol-3-phosphate O-acyltransferase 1 |
| AKT1S1 | AKT1 substrate 1 |
| APOB | apolipoprotein B |
| APOC3 | apolipoprotein C3 |
| APOE | apolipoprotein E |
| ARG1 | arginase 1 |
| BDH1 | 3-hydroxybutyrate dehydrogenase 1 |
| CREBBP | CREB binding protein |
| CXCL8 | C-X-C motif chemokine ligand 8 |
| DEPTOR | DEP domain containing MTOR interacting protein |
| DGAT1 | diacylglycerol O-acyltransferase 1 |
| ELOVL2 | ELOVL fatty acid elongase 2 |
| ELOVL5 | ELOVL fatty acid elongase 5 |
| FABP1 | fatty acid binding protein 1 |
| FADS1 | fatty acid desaturase 1 |
| FADS2 | fatty acid desaturase 2 |
| FGF19 | fibroblast growth factor 19 |
| GCKR | glucokinase regulator |
| GNMT | glycine N-methyltransferase |
| IL10 | interleukin 10 |
| IL1B | interleukin 1 beta |
| IL6 | interleukin 6 |
| INS | insulin |
| LPCAT1 | lysophosphatidylcholine acyltransferase 1 |
| LYPLAL1 | lysophospholipase like 1 |
| MBOAT7 | membrane bound O-acyltransferase domain containing 7 |
| MIR122 | microRNA 122 |
| MIR141 | microRNA 141 |
| MIR200C | microRNA 200c |
| MT-CO2 | mitochondrially encoded cytochrome c oxidase II |
| MTOR | mechanistic target of rapamycin kinase |
| MTTP | microsomal triglyceride transfer protein |
| NCAN | neurocan |
| NFE2L2 | "nuclear factor, erythroid 2 like 2" |
| NLRP3 | NLR family pyrin domain containing 3 |
| NOS2 | nitric oxide synthase 2 |
| NR5A2 | nuclear receptor subfamily 5 group A member 2 |
| NR5A2 | nuclear receptor subfamily 5 group A member 2 |
| PEMT | phosphatidylethanolamine N-methyltransferase |
| PNPLA3 | patatin like phospholipase domain containing 3 |
| PPARA | peroxisome proliferator activated receptor alpha |
| PPARG | peroxisome proliferator activated receptor gamma |
| PPP1R3B | protein phosphatase 1 regulatory subunit 3B |
| PRKAA1 | protein kinase AMP-activated catalytic subunit alpha 1 |
| PRKAA2 | protein kinase AMP-activated catalytic subunit alpha 2 |
| PTGS2 | prostaglandin-endoperoxide synthase 2 |
| RETN | resistin |
| SLC27A1 | solute carrier family 27 member 1 |
| SREBF1 | sterol regulatory element binding transcription factor 1 |
| TGFB1 | transforming growth factor beta 1 |
| TLR2 | toll like receptor 2 |
| TLR4 | toll like receptor 4 |
| TM6SF2 | transmembrane 6 superfamily member 2 |
| TMC4 | transmembrane channel like 4 |
| TNF | tumor necrosis factor |
